# The emerging role for neutrophil mitochondrial metabolism in lung inflammation

**DOI:** 10.1097/IN9.0000000000000036

**Published:** 2024-01-25

**Authors:** Mary E. Maldarelli, Michael J. Noto

**Affiliations:** 1 Division of Pulmonary and Critical Care Medicine, Department of Medicine, University of Maryland School of Medicine, Baltimore, MD, USA

**Keywords:** neutrophil, mitochondrial metabolism, lung, pulmonary, inflammation, infection

## Abstract

Recent advances shed light on the importance of mitochondrial metabolism in supporting essential neutrophil functions such as trafficking, NETosis, bacterial killing, and modulating inflammatory responses. Mitochondrial metabolism is now recognized to contribute to a number of lung diseases marked by neutrophilic inflammation, including bacterial pneumonia, acute lung injury, and chronic obstructive pulmonary disease. In this mini review, we provide an overview of neutrophil metabolism focusing on the role of mitochondrial programs, discuss select neutrophil effector functions that are directly influenced by mitochondrial metabolism, and present what is known about the role for mitochondrial metabolism in lung diseases marked by neutrophilic inflammation.

## 1. Introduction

Neutrophils are the most abundant leukocytes found in the peripheral blood of humans ^[1]^, respond rapidly to infection, inflammation, and tissue damage ^[2]^, and function as a first line of defense ^[3]^. Neutrophils play a critical role in combating infection with a multitude of specialized effector functions that contain and kill pathogens, including the phagocytosis of microbes, the generation of toxic reactive oxygen species (ROS) through the assembly and activation of the nicotinamide adenine dinucleotide phosphate (NADPH) oxidase, the release of antimicrobial contents of cytoplasmic granules via degranulation, and the extrusion of neutrophil extracellular traps (NETs) that trap and kill pathogens through a process known as NETosis ^[3,4]^. The importance of neutrophils in this context is highlighted by the marked susceptibility to infection in persons with neutropenia ^[5,6]^ or defects in neutrophil effector functions, such as defective NADPH oxidase in persons with chronic granulomatous disease ^[7,8]^. The functions that neutrophils employ to destroy pathogens can also damage host tissue, perpetuate inflammation, and cause organ dysfunction ^[9,10]^. Neutrophils are now recognized to play a central role in the pathogenesis and progression of a number of inflammatory conditions ^[11]^, autoimmune diseases ^[12]^, and cancer ^[13]^. Therefore, the pivotal role of neutrophils in human health and numerous disease states is well established.

Neutrophils have historically been considered as terminally-differentiated, homogeneous, short-lived cells that are continuously produced in high numbers by the bone marrow ^[1]^ and rely on glycolysis to fuel their cellular functions ^[14,15]^. It is now recognized that neutrophils are longer lived in tissue ^[16]^, respond to environmental cues with active transcriptional and translational programs ^[17–19]^, are phenotypically heterogeneous in homeostasis and disease states ^[20–23]^, have the considerable capacity to regulate immune function ^[24]^ and tissue repair ^[25,26]^, and possess greater metabolic complexity than was previously recognized. In this mini review, we will focus on mitochondrial metabolic programs in neutrophil biology with an emphasis on their role in neutrophilic lung inflammation. Comprehensive reviews of neutrophil metabolism and the roles of mitochondria in neutrophils are found elsewhere ^[27,28]^.

## 2. Neutrophil metabolism

**Figure 1.**
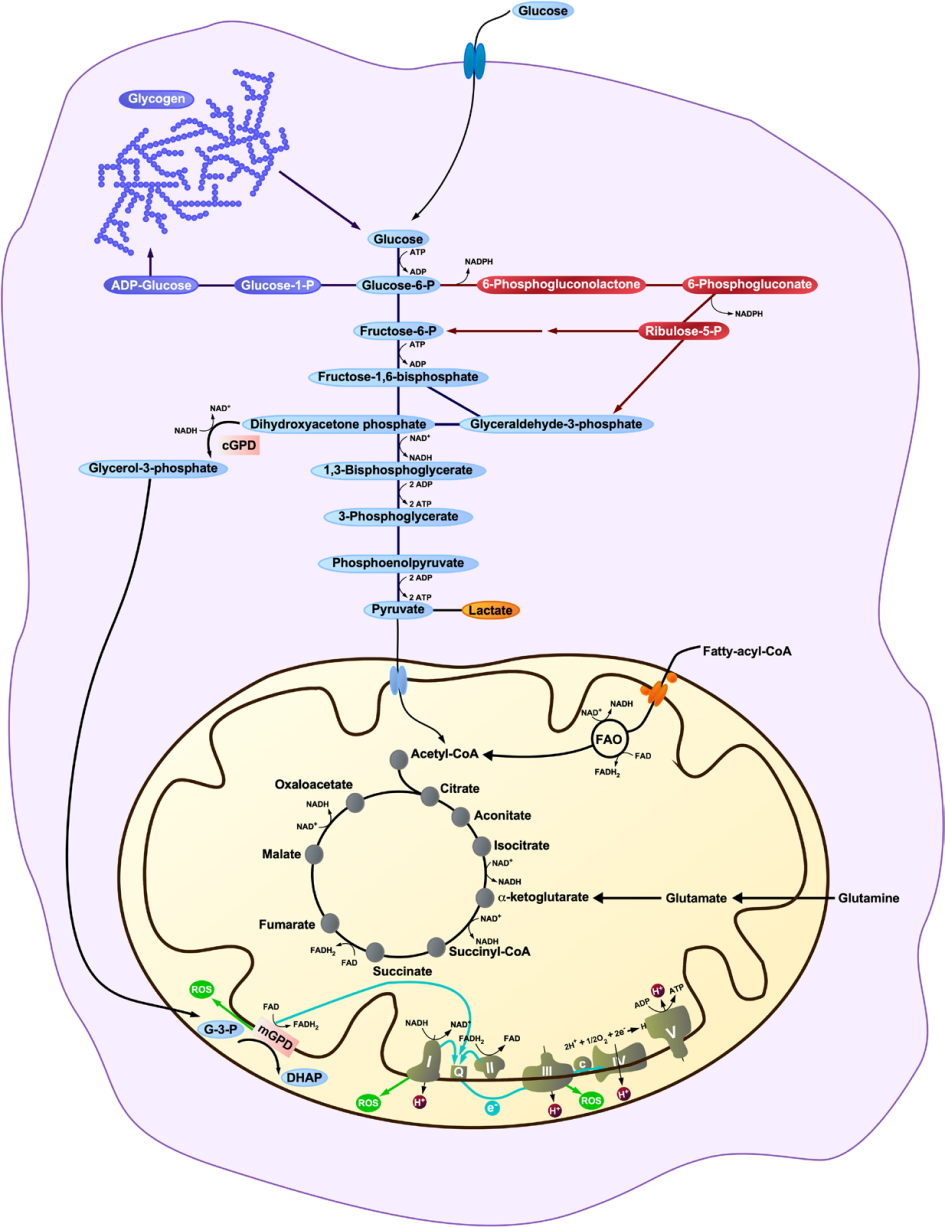
**An overview of neutrophil metabolism.** The cytosolic metabolic programs of glycolysis (light blue), glycogenesis/glycogenolysis (purple), and pentose phosphate pathway (red) are shown as well as the mitochondrial programs of fatty acid β-oxidation, glutaminolysis, TCA cycle, glycerol-3-phosphate shuttle, and the electron transport chain. TCA, tricarboxylic acid.

Glucose metabolism has long been recognized as the central metabolic program in neutrophils ^[15,29]^. Neutrophil expression of glucose transporters GLUT1, GLUT3, and GLUT4 on the plasma membrane is relatively high under resting conditions and expression increases further upon insulin exposure or neutrophil activation, thereby facilitating glucose uptake ^[30,31]^. In addition, neutrophils contain glycogen stores ^[15,29]^ that are increased upon activation or under conditions of hypoxemia and provide a ready source of glucose to fuel effector functions that require a rapid increase in glycolytic flux or when glucose levels are low or absent in the extracellular environment ^[32–34]^. Flux through glycolysis is high in neutrophils and accounts for the vast majority of cellular adenosine triphosphate (ATP) produced under resting and activated conditions, is further increased during activation, and fuels neutrophil effector functions that include phagocytosis, degranulation, and random migration ^[15,31,34,35]^. The vast majority of pyruvate generated from glycolysis is converted to lactate, with relatively little available to enter the mitochondria ^[15]^. The oxidative burst requires significant quantities of NADPH to power the NADPH oxidase (NOX). Upon activation, the required increase in NADPH production is accomplished by a rapid shift from lower glycolysis to the pentose phosphate pathway (PPP) that is mediated by relief of the cellular NADP^+^ limitation that prevents the PPP from reaching its capacity. As NADPH is consumed by the NADPH oxidase, the resulting NADP^+^ allows flow preferentially through the PPP, which generates NADPH, thus facilitating superoxide production by NOX ^[36,37]^. NADP^+^ availability facilitates the conversion of glucose-6-phosphate to 6-phosphogluconate and ribulose-5-phospate in the PPP, resulting in NADPH production. Ribulose-5-phosphate is converted to fructose-6-phospate, which is then converted back to glucose-6-phosphate by the glycolytic enzyme phosphoglucose isomerase. This glucose cycling through the PPP and upper glycolysis facilitates maximal NADPH production from a single molecule of glucose by diverting glucose metabolism from lower glycolysis and pyruvate generation to the PPP ^[37]^. Activation of NETosis involves increased intracellular ROS that may be mediated by NOX. Therefore, both glycolysis and the PPP contribute to NOX-dependent NETosis ^[38–40]^. In contrast to many cell types, the energy for the majority of cellular processes and effector functions in neutrophils is provided by the cytoplasmic programs of glycolysis, glycogenolysis, and the PPP. As a result, the role of mitochondrial metabolism in neutrophil biology was largely underexplored. It is now clear that mitochondrial metabolism plays important roles in neutrophils (Figure 1).

Although less numerous than in other cell types ^[41]^, neutrophil mitochondria are distributed throughout the cytoplasm as a rich network. Neutrophil mitochondria have a polarized inner membrane and this membrane potential can be dissipated by the uncoupling agent carbonyl cyanide-*p*-trifluoromethoxyphenylhydrazone (FCCP), resulting in changes to neutrophil cell morphology ^[42]^. An active mitochondrial membrane potential is indicative of electron transfer to the electron transport chain (ETC), resulting in the pumping of hydrogen ions across the inner mitochondrial membrane by complexes I (nicotinamide adenine dinucleotide [NADH]-ubiquinone oxidoreductase), III (ubiquinone-cytochrome C oxidoreductase), and/or IV (cytochrome C oxidase). Complex IV catalyzes the final transfer of electrons to oxygen resulting in the formation of water ^[43]^. However, the mitochondrial respiratory contribution to oxygen consumption in mature neutrophils is minimal ^[29,44,45]^, suggesting that neutrophils are less reliant on complex IV activity and oxygen as the terminal electron acceptor. In addition, mitochondrial-derived ATP production is modest, contributing no more than 10%–15% of the total cellular ATP pool ^[15,34,46]^. In support of this, human neutrophils express low levels of complex IV with markedly reduced complex IV activity relative to peripheral blood mononuclear cells (PBMCs) ^[46]^. While the activity of complexes II and V in neutrophils is comparable to PBMCs, the activity of complexes I and III is reduced. Inhibition of complex III lowers the mitochondrial membrane potential to a greater extent than inhibition of complexes I or IV, suggesting that membrane polarization results primarily from complex III activity ^[46]^. The low complex IV activity may enable electron leak from complex III and to a lesser extent complex I, resulting in mitochondrial superoxide generation ^[47]^. Furthermore, the ETC in neutrophils lacks respiratory supercomplex organization ^[46]^. Respiratory supercomplexes contribute to the efficient coupling of the respiratory chain to ATP synthesis and their absence in neutrophils likely impacts both ATP generation and oxygen consumption. The unique structure of the ETC coupled with the reliance on glycolysis for cellular energy requirements renders neutrophils uniquely pre-programmed to function in conditions of low environmental oxygen content, whereas macrophages undergo ETC adaptation only after interacting with a pathogen ^[48]^. These metabolic characteristics of mature neutrophils differ from those of neutrophil precursors, immature neutrophils, and low-density neutrophils, which are more reliant on mitochondrial metabolism and oxidative phosphorylation ^[49–52]^.

The tricarboxylic acid (TCA) cycle generates NADH and dihydroflavine-adenine dinucleotide (FADH_2_) that each transfer electrons to the ETC. Pyruvate derived from glycolysis can enter the mitochondria and be converted to acetyl-coenzyme A (acetyl-CoA) by the pyruvate–dehydrogenase complex. Acetyl-CoA enters the TCA cycle via conversion to citrate by the TCA enzyme citrate synthase ^[43]^. As mentioned above, the majority of cellular pyruvate is converted to lactate in mature neutrophils ^[15]^ and there is little experimental evidence to indicate that glucose-derived pyruvate is an important substrate for neutrophil mitochondria. Acetyl-CoA is also generated from long-chain fatty acids via mitochondrial β-oxidation (FAO) ^[53]^. Carbon labeling experiments have demonstrated that FAO is active in neutrophils, contributes substrate to the TCA cycle, and is reduced by neutrophil activation by lipopolysaccharide (LPS) ^[34,54]^. In addition, the iterative process of FAO generates NADH and FADH_2_, which may transfer electrons directly to the ETC ^[53]^. FAO contributes to maintenance of the mitochondrial membrane potential as inhibition of FAO has been demonstrated to reduce the membrane potential ^[54]^. Glutamine metabolism through glutaminolysis produces α-ketoglutarate, an intermediate in the TCA cycle ^[43]^. Under glucose-limited conditions, lung neutrophils take up extracellular proteins for glutamine scavenge, which is metabolized and serves as a substrate for the TCA cycle ^[18]^. Mitochondrial metabolism can also contribute intermediates for biosynthetic reactions. Neutrophils are now recognized to have active gluconeogenesis and glycogen synthesis programs that contribute to glucose and glycogen stores. These processes are fueled, in part, by glutaminolysis and the TCA cycle ^[34]^. TCA-derived malate can be an important source of NADPH, which has been demonstrated in malignant cells ^[55]^, and may contribute to the high NADPH requirements of neutrophils.

While glucose-derived pyruvate has not been demonstrated to contribute significantly to mitochondrial metabolism, an alternative link between glycolysis and the ETC exists in the form of the glycerol-3-phosphate (G-3-P) shuttle. Glycolysis generates NADH. To maintain high flux through glycolysis, NAD^+^ must be regenerated. This is accomplished through the reduction of pyruvate to lactate by lactate dehydrogenase and by the reduction of dihydroxyacetone phosphate (DHAP) to G-3-P by cytosolic NAD^+^-linked glycerol-3-phosphate dehydrogenase (GPD) ^[56]^. Both processes are active in neutrophils. G-3-P can diffuse into the mitochondria where it is converted to DHAP by the flavin adenine dinucleotide (FAD)-linked mitochondrial GPD. Electrons from the resulting FADH_2_ can enter the ETC. The addition of exogenous glycerol phosphate to isolated neutrophil mitochondria rescues the membrane potential to a greater extent than the addition of glutamate or succinate ^[46]^. Inhibition of mitochondrial GPD in neutrophils has been demonstrated to reduce the mitochondrial membrane potential ^[57]^, indicating that the G-3-P shuttle is active in neutrophils, contributes to the ETC, and maintains the mitochondrial membrane potential in combination with the TCA cycle. While much remains to be learned about neutrophil metabolism, it is clear that neutrophils rely on uniquely specialized metabolic programs to support their multiple functions in the context of health and disease.

## 3. Environmental metabolic shifts in the lung

**Figure 2.**
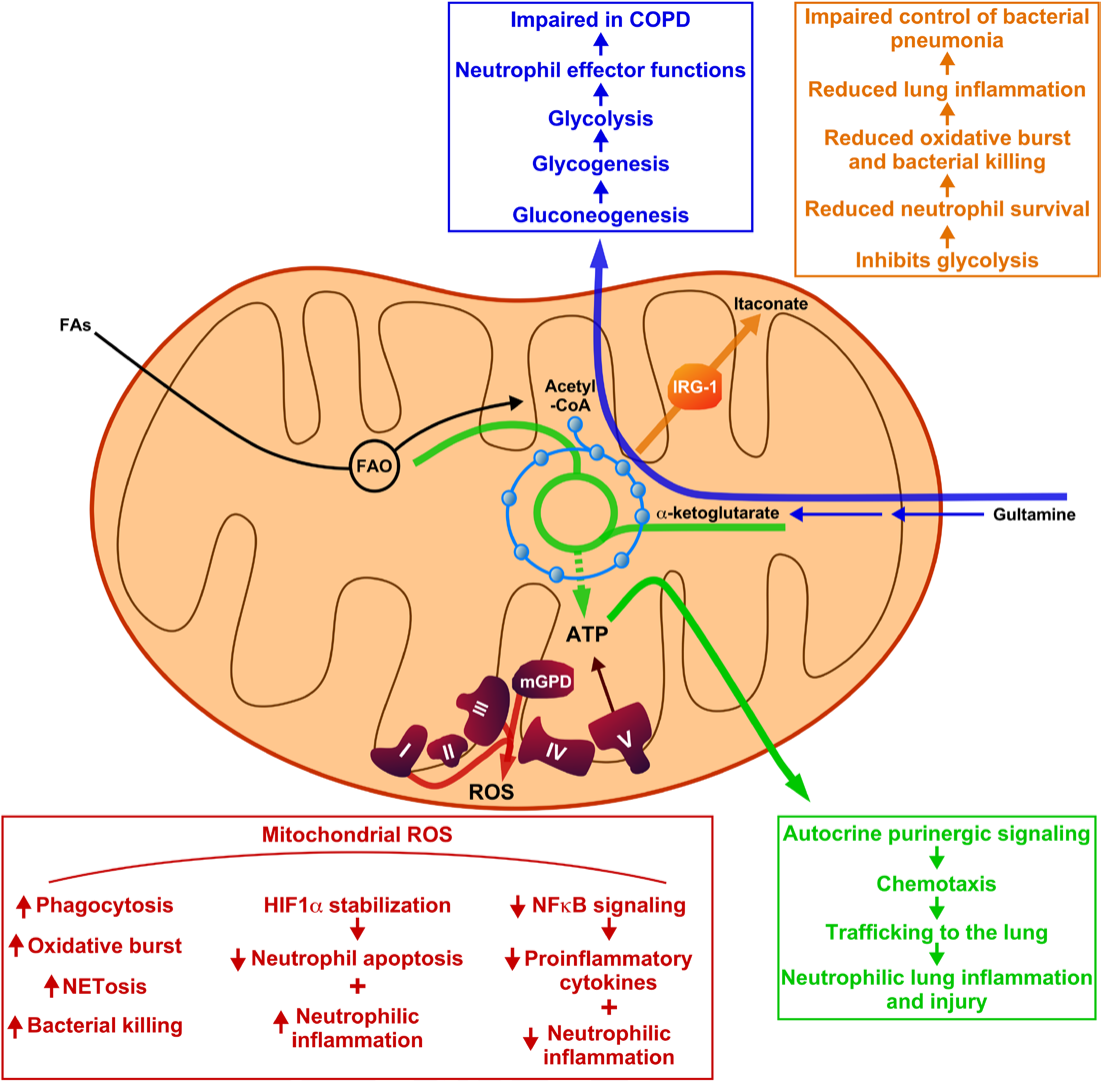
**The influence of mitochondrial metabolism on neutrophilic lung inflammation.** Mitochondrial ATP (green), ROS (red), itaconate production (orange), and mitochondrial-derived substrates for gluconeogenesis (blue) all contribute to neutrophil effector functions relevant to inflammatory lung diseases. ROS, reactive oxygen species.

Much of what is known about neutrophil metabolic programs is derived from ex vivo manipulation of human blood or mouse neutrophils in oxygen and nutrient-rich conditions. However, environmental metabolic conditions differ in the lung. Glucose is central to neutrophil metabolism and glucose availability in the lung may be best approximated by airway surface liquid (ASL). ASL is a sterile liquid with low glucose levels and low pH that restricts the growth of pathogens. Differential polarization of glucose transporters GLUT1 and GLUT10 to the lung epithelial basolateral and apical membranes, respectively, maintains ASL glucose at low levels ^[58,59]^. ASL glucose levels fluctuate with inflammation as LPS-treated mice exhibit an approximate 9-fold increase in glucose concentration in the trachea ^[60]^. The restriction of glucose in the ASL functions to limit bacterial growth ^[58]^ and an increase of glucose in ASL has been associated with proliferation of pathologic bacteria ^[61]^. The lung presents an oxygen-rich environment under homeostatic conditions but lung hypoxia may develop in response to altitude exposure or regional variations in lung ventilation, perfusion, or oxygen consumption in the context of inflammation. Hypoxia is associated with increased morbidity and mortality in mouse pneumonia models ^[62]^ and in humans with pneumonia ^[63]^. Inflammation-induced lung injury is characterized by the influx of inflammatory edema fluid into the lung airspaces. This edema fluid is metabolically distinct, is rich in protein and lipids, and presents a marked shift in substrate availability for immune cells recruited to the lungs in response to inflammatory or injurious stimuli ^[64]^. Therefore, the metabolic programs active in neutrophils during optimized in vitro culture conditions are unlikely to fully recapitulate metabolism in the inflamed lung (Figure 2).

## 4. Mitochondrial metabolism in chemotaxis and trafficking

While the energy requirements for phagocytosis, degranulation, oxidative burst, NETosis, and random migration depend upon glycolysis, neutrophil chemotaxis was found to be resistant to the inhibition of glycolysis ^[35]^. Chemotaxis was later found to depend upon mitochondrial metabolism as either dissipation of the mitochondrial membrane potential with FCCP or inhibition of mitochondrial ATP synthesis with oligomycin was found to impair neutrophil chemotaxis ^[42]^. The mechanistic link between mitochondrial bioenergetics and neutrophil chemotaxis involves amplification of chemoattractant signals through autocrine purinergic signaling ^[65,66]^. Treatment of neutrophils with the peptide *N*-formyl-Met-Leu-Phe (fMLF) or activators of other G-protein-coupled chemoattractant receptors induces intracellular Ca^2+^ flux ^[67]^ and localization of mitochondria adjacent to pannexin-1 channels in the plasma membrane ^[68,69]^. The initial wave of intracellular Ca^2+^ triggers mitochondria to release ATP, which then passes outside the cell through pannexin-1 ^[70]^. This extracellular ATP of mitochondrial origin is available to activate the P2Y2 receptor in an autocrine fashion ^[70,71]^, which results in a second wave of intracellular Ca^2+^ flux and subsequent activation of MAP kinase cascades necessary for essential neutrophil functions, including chemotaxis ^[67–71]^. Mitochondrial-derived ATP is required for maximal intracellular Ca^2+^ flux as disrupting mitochondrial ATP production with FCCP impairs autocrine purinergic signaling and chemotaxis ^[70,71]^. Therefore, the energy metabolism of neutrophil mitochondria facilitates the signaling involved in neutrophil chemotaxis and activation.

Neutrophil chemotaxis is critical for neutrophil trafficking to sites of inflammation or tissue damage. To this end, inhibition of mitochondrial complex I or III with rotenone or antimycin, respectively, and dissipating the mitochondrial membrane potential with FCCP disrupted neutrophil trafficking at the organismal level in a zebrafish model ^[72]^. We have demonstrated that disruption of mitochondrial FAO impairs mitochondrial bioenergetics in neutrophils and reduces autocrine purinergic signal amplification, chemotaxis, and neutrophil trafficking to the lungs in the context of bacterial pneumonia. FAO inhibition resulted in a marked reduction in neutrophil accumulation in the lung, reduced inflammation, impaired clearance of multiple bacterial pathogens, and decreased survival in a mouse model of bacterial pneumonia ^[54]^. Therefore, mitochondrial metabolism and bioenergetics are fundamental to appropriate neutrophil trafficking to the lungs in the context of an inflammatory stimulus and disrupting these processes diminishes neutrophilic lung inflammation.

## 5. Mitochondrial ROS

Mitochondrial ROS are generated from the ETC by transfer of electrons to oxygen by respiratory complexes I and III to form superoxide ^[47]^. Electron leak from mitochondrial GPD and the G-3-P shuttle also contributes to mitochondrial ROS production ^[73]^. In neutrophils, scavenging mitochondrial ROS reduces phagocytosis and oxidative burst ^[74]^, and mitochondrial ROS facilitate NETosis induced by certain activating signals ^[75]^. Mitochondrial ROS directly contributes to killing of *Streptococcus pneumoniae* by neutrophils and scavenging mitochondrial ROS impairs *S. pneumoniae* killing to a greater extent than inhibition of the NADPH oxidase ^[76]^. In a zebrafish larvae model of *Salmonella enterica* infection, neutrophils exhibit trained immunity that protects from subsequent infection. Neutrophils derived from infection-experienced hematopoietic stem and progenitor cells have increased mitochondrial mass, have increased mitochondrial ROS production, and demonstrate enhanced bacterial killing that is mitochondrial ROS dependent ^[77]^. Through the generation of ROS, mitochondrial metabolism contributes directly to fundamental neutrophil effector functions.

Mitochondrial ROS function as important signaling molecules. ROS generated by mitochondrial GPD stabilize subunit 1 alpha of the hypoxia inducible factor transcription factor (HIF-1α) in neutrophils and inhibition of mitochondrial GPD reverses the resulting increase in HIF-1α abundance ^[57]^. HIF effector genes regulate anaerobic metabolism, HIF-1α stabilization increases neutrophil survival by preventing apoptosis ^[78]^, and HIF-1α is essential for inflammation mediated by myeloid cells ^[79]^. Increased HIF-1α resulting from myeloid-specific inactivation of prolyl hydroxylase 2, an enzyme that targets HIF-1α for degradation, results in increased neutrophil chemotaxis and increased neutrophil recruitment to the lungs. These exaggerated neutrophil responses result in more severe lung injury in a *S. pneumoniae* mouse model of bacterial pneumonia ^[80]^. In a similar mouse pneumonia model, hypoxia-induced stabilization of HIF-1α increased morbidity and mortality during intratracheal *S. pneumoniae* infection ^[62]^. Therefore, mitochondrial ROS-mediated stabilization of HIF-1α likely contributes to exaggerated neutrophilic responses and links mitochondrial metabolism with neutrophilic lung inflammation in the context of pneumonia. In contrast to the pro-inflammatory effects of mitochondrial ROS mediated by HIF stabilization, mitochondrial ROS interfere with nuclear factor kappa B (NFкB) signaling in neutrophils. Inhibition of complexes I and III in neutrophils increased intracellular superoxide and hydrogen peroxide formation. The increased mitochondrial ROS impaired NFкB signaling in response to LPS and reduced pro-inflammatory cytokine production by neutrophils. This process appears to depend on hydrogen peroxide since catalase reversed these phenotypes. In a mouse model of LPS-induced lung injury, systemic treatment of mice with either complex I or complex III inhibitors reduced pro-inflammatory cytokine production in the lung, neutrophil recruitment to the lung, and lung injury ^[81,82]^.

## 6. Mitochondrial metabolism supports glycolysis

As noted above, neutrophils contain glycogen stores and the dynamic between glycogenolysis and uptake of extracellular glucose varies by environmental glucose availability and the different effector functions activated in neutrophils. Glycogenesis is fueled, in part, by active gluconeogenesis via the mitochondrial metabolic programs of glutaminolysis and FAO ^[34]^. In glucose-deplete conditions, neutrophils take up extracellular proteins, including albumin and IgM, which are degraded in the lysosome and provide amino acid substrates for glutaminolysis, which enter the TCA cycle and central carbon metabolism ^[18]^. Inhibiting glutaminolysis in neutrophils reduces glycogen stores and impairs bacterial killing ^[34]^. Neutrophils from patients with chronic obstructive pulmonary disease (COPD) – a disease marked by chronic neutrophilic airway inflammation – demonstrate an impaired ability to increase cellular glycogen in response to LPS stimulation, reduced glycolytic capacity, and a reduced capacity to use glutamine as a substrate for gluconeogenesis. This results in impaired neutrophil killing of *S. pneumoniae* and *Staphylococcus aureus* ^[34]^. This work demonstrates the importance of mitochondrial metabolism in providing metabolic flexibility to fuel glycogenesis and glycolysis when glucose is limited. Impairment of this ability is associated with defective neutrophilic responses in persons with COPD that may contribute to neutrophilic airway inflammation and lung dysfunction.

## 7. Metabolic modulation of neutrophilic inflammation

The TCA cycle intermediate aconitate can be diverted from the TCA cycle and converted to itaconate by the enzyme immune-responsive gene 1 protein (IRG-1), which is also known as aconitate decarboxylase 1 ^[83]^. Expression of IRG-1 is induced by numerous inflammatory stimuli, including LPS, and IRG-1 localizes to mitochondria ^[84]^. Itaconate targets multiple cellular pathways resulting in net anti-inflammatory effects by inhibiting glycolysis ^[85]^, inhibiting inflammasome activation ^[86]^, inhibiting succinate dehydrogenase ^[87]^, and inhibiting Janus kinase (JAK) signaling ^[88]^. Myeloid cells in the lung produce high levels of itaconate in response to bacterial infections caused by *Pseudomonas aeruginosa* ^[89]^, *Klebsiella pneumoniae* ^[90]^, and *S. aureus* ^[91]^. Unlike the Gram-negative pathogens, *S. aureus* induces itaconate production primarily by neutrophils in the lung and pulmonary itaconate levels are reduced significantly by neutrophil depletion in *S. aureus*-infected mice. In neutrophils, itaconate inhibits glycolysis, which reduces neutrophil survival, neutrophil oxidative burst, and *S. aureus* killing *in vitro. In vivo*, this manifests by impaired bacterial clearance from the lung in *S. aureus*-infected mice. The impaired bacterial clearance is reversed in mice lacking IRG-1 ^[91]^. This exemplifies how neutrophil mitochondrial metabolism can be reprogrammed to limit neutrophilic lung inflammation in the context of bacterial pneumonia.

## 8. Conclusions

Despite nearly 7 decades of interest in the metabolic requirements for neutrophil effector functions, the past 10 years have marked a significant increase in the understanding of mitochondrial metabolic contributions to neutrophil biology. It is now clear that the role for mitochondria in neutrophils extends well beyond mediating apoptosis and mitochondrial metabolism is relevant to a number of inflammatory lung diseases. Mitochondrial metabolic programs are required for neutrophil chemotaxis and trafficking to sites of infection or inflammation; directly contribute to bacterial killing, NETosis, and signaling; facilitate glucose metabolism; and the redirection of mitochondrial metabolism limits inflammation. These programs influence neutrophilic inflammation and disease progression in bacterial pneumonia, COPD, and acute lung injury. As neutrophilic inflammation plays a role in myriad additional lung diseases including asthma, fibrosis, cancer, and bronchiectasis, the current understanding of mitochondrial metabolism in neutrophilic lung inflammation likely greatly underestimates the importance of these programs in pulmonary diseases.

## Conflicts of interest

M.E.M. has no conflicts of interest. M.J.N. has no conflicts of interest.

## Funding

M.J.N. is funded by National Institutes of Health award 1R01HL152210-03 to M.J.N.

## Acknowledgments

M.E.M. and M.J.N. designed this project, wrote the manuscript, and have primary responsibility for the content. All authors have read and approved the final manuscript.
